# First Report on High Entropy Alloy Nanoparticle Decorated Graphene

**DOI:** 10.1038/s41598-018-27096-8

**Published:** 2018-06-07

**Authors:** M. Y. Rekha, Nitin Mallik, Chandan Srivastava

**Affiliations:** Department of Materials Engineering, Indian Institute of Science, Bangalore, 560012 India

## Abstract

This is the first report on synthesis of multimetal high entropy alloy (HEA) nanoparticle-few layer graphene composite. A two-step methodology for synthesizing multi-component HEA nanoparticle-graphene composite is provided. In the first step, high purity graphite powder was mechanically milled with metal powders (Ni, Cr, Co, Cu, Fe) to produce multimetal-graphite composite. This composite was then sonicated with sodium lauryl sulphate (SLS) for 2 hours to produce a dispersion of graphene decorated with multi-component nanoparticles with face centred cubic structure. Potentiodynamic polarization and electrochemical impedance spectroscopy methods revealed that the HEA nanoparticle graphene composite possess excellent corrosion resistance properties which was better than the corrosion resistance exhibited by milled and exfoliated graphene. The HEA nanoparticle-graphene composite can be used for corrosion resistant coating applications.

## Introduction

Synthesis, characterization and investigation of properties of multi-component solids have gained considerable attention in the recent years. These multi-component alloys, also referred as high entropy alloys (HEAs), contain five or more component metal atoms in a single phase solid solution structure. The preference to form solid solution over intermetallic compounds between component metal atoms is essentially due to the high configurational entropy of the solid solution phase^1–3^. Several reports have illustrated the relevance of factors such as high configurational entropy, lattice distortion, sluggish diffusion and cocktail effect in high entropy alloys^1–6^. HEAs exhibit several useful properties such as high corrosion resistance, high wear resistance, high strength and excellent oxidation resistance^7–12^.

Graphene is a two-dimensional material composed of carbon atoms packed through sp^2^ hybridization^13^. Graphene has attracted significant attention due to its extraordinary mechanical^14^, thermal^15^, optical^16^ and electronic properties^17^. In addition to graphene, graphene based composites are now also being extensively produced and investigated due to their unique properties^18^. Common methods used for synthesizing graphene reinforced metal/polymer matrix composites are electrodeposition technique^19,20^ which involves dispersion of graphene in the electrolyte bath used for electrodeposition, powder metallurgy^21,22^ technique in which graphene is initially mixed with mechanically milled powder followed by compaction and sintering of the polymer/metal powder-graphene mixture, stir casting^23^ technique in which graphene is added into molten metal or polymer slurry under stirring condition followed by casting in a mold. Common methods used for synthesizing graphene based composites in which graphene forms the support over which nano-sized solids are attached are mechanical-milling^24–28^, solvothermal process^29^, hydrothermal process^30,31^, two-steps hydrothermal and solvothermal process^32^, polyol method^33^, sol-gel method^34^, pulsed laser ablation^35^, *in-situ* electrochemical polymerization method^36^, *ex-situ* method involving attachment of metallic nanoparticles to the surface of graphene sheets by covalent or non-covalent interactions^37^.

There is no report in the literature on the synthesis and characterization of graphene-multi-component HEA nanoparticle composites. Saiphaneendra *et al*.^38^ have used mechanical milling approach to synthesize multi-component deposits on the graphene surface. These multi-component deposits are however not nanoparticles. In another report, Singh *et al*.^39^ have illustrated a wet chemical synthesis based methodology to produce only isolated multi-component nanoparticles without graphene support. In another report, Lu *et al*.^40^ have provided solidification based methodology for synthesizing bulk HEA alloys with ultra-fine grain sizes but without graphene incorporation.

This report provides a methodology for synthesizing multi-component HEA nanoparticle-graphene composite using the mechanical milling and sonication assisted exfoliation approach. The multi-component nanoparticle system produced here is NiFeCrCoCu high entropy alloy. Corrosion properties of this composite was also evaluated in the present work.

## Methods

In the present work, a two-step approach was used. In the first step, multimetal-graphite composite was produced and in the second step, this composite was subjected to sonication based exfoliation to produce multi-metal HEA nanoparticle decorated graphene.

Multimetal-graphite composite was produced by using the mechanical milling technique^41^. High purity graphite rods were initially grinded in mortar. 4 g of the graphite powder was then mixed with elemental blend of 0.193 g of iron, 0.180 g of chromium, 0.204 g of cobalt, 0.220 g of copper and 0.203 g of nickel powders. This mixture was then transferred to a hard chromium steel vial containing stainless steel balls. Ball-to-powder weight ratio was 20:1. Mechanical milling of this mixture was then done in toluene medium for 80 hours. After milling, the mixture was dispersed in a solution of 10 ml ethanol and 20 mg sodium lauryl sulphate (SLS) followed by sonicated for 2 hrs for the exfoliation of graphene. After sonication, the dispersion was subjected to centrifugation at 8000 rpm in order to remove the SLS. The centrifuged powder was then dispersed in ethanol and allowed to settle for about 5–6 hrs. The resultant supernatant solution containing graphene-nanoparticle composite was then isolated.

X-pert pro X-ray diffractometer with a Cu K_α_ radiation source was used for x-ray diffraction (XRD) analysis. Scanning electron microscope (SEM) (ESEM Quanta-200), with energy dispersive X-ray spectroscopy (EDS) detector was used for imaging and elemental compositional analysis. UV-Visible absorption spectroscopic experiments were carried out in 700 to 200 nm wavelength range using Perkin Elmer (Lambda 35) UV-Vis Spectrometer. Raman spectrum were recorded using a setup (HORIBA JOBIN YVON, Lab RAM HR) consisting of Diode-pumped solid-state laser operating at 532 nm with a charge coupled detector. A 300 kV FEI TITAN transmission electron microscope (TEM) was used for imaging the samples and for obtaining selected area electron diffraction (SAED) patterns from them. Scanning transmission electron microscopy-energy dispersive spectroscopy (STEM-EDS) technique in TITAN TEM was used for compositional analysis of the samples. Atomic force microscopy (AFM) experiments were carried out at room temperature using a Nanosurf AFM instrument (Switzerland). Corrosion properties were measured using a conventional three electrode cell setup and a CHI 604 electrochemical workstation. The coated sample (produced by coating the mixture of HEA nanoparticle-graphene composite and Araldite epoxy over mild steel) of 1 cm^2^ exposed area, platinum foil and Ag/AgCl electrode were used as a working, counter/auxiliary and reference electrodes respectively.

## Result and Discussion

**Figure 1:**
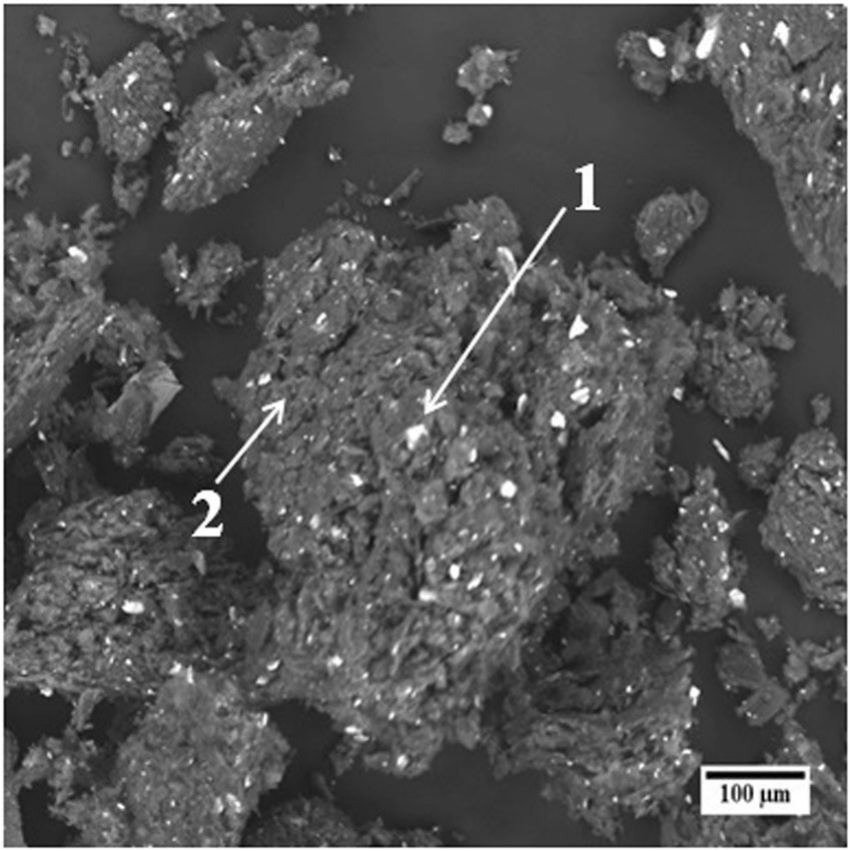
SEM back-scattered electron micrograph of 80 hours milled sample. Graphite (2) and multimetal clusters (1) are denoted by arrows in the SEM micrograph.

**Figure 2:**
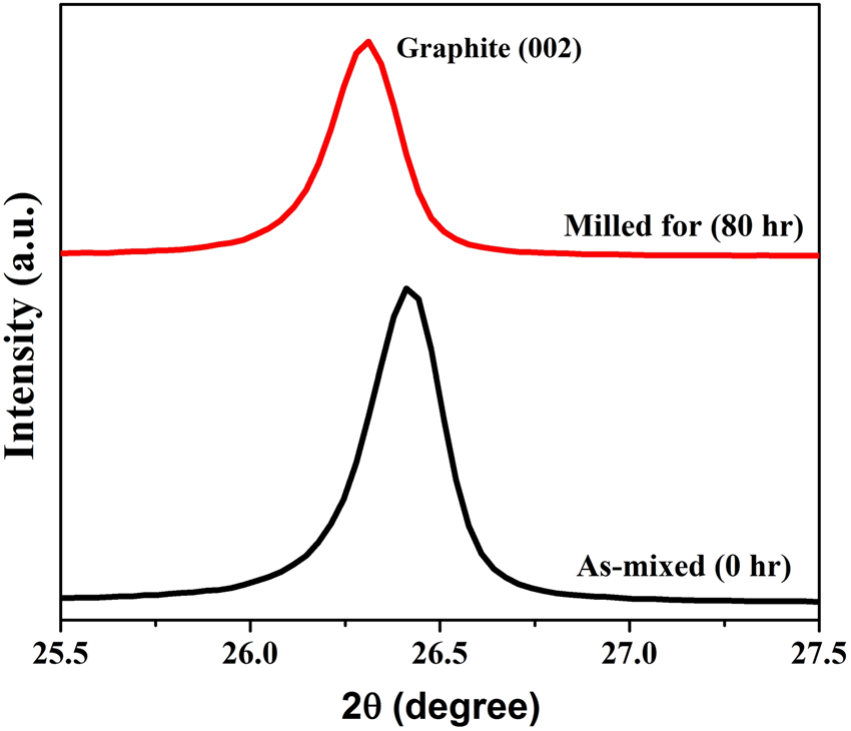
X-ray diffraction (XRD) profiles, showing the graphite peak, obtained from as-mixed powder (zero hours milling) and powder sample milled for 80 h.

**Figure 3:**
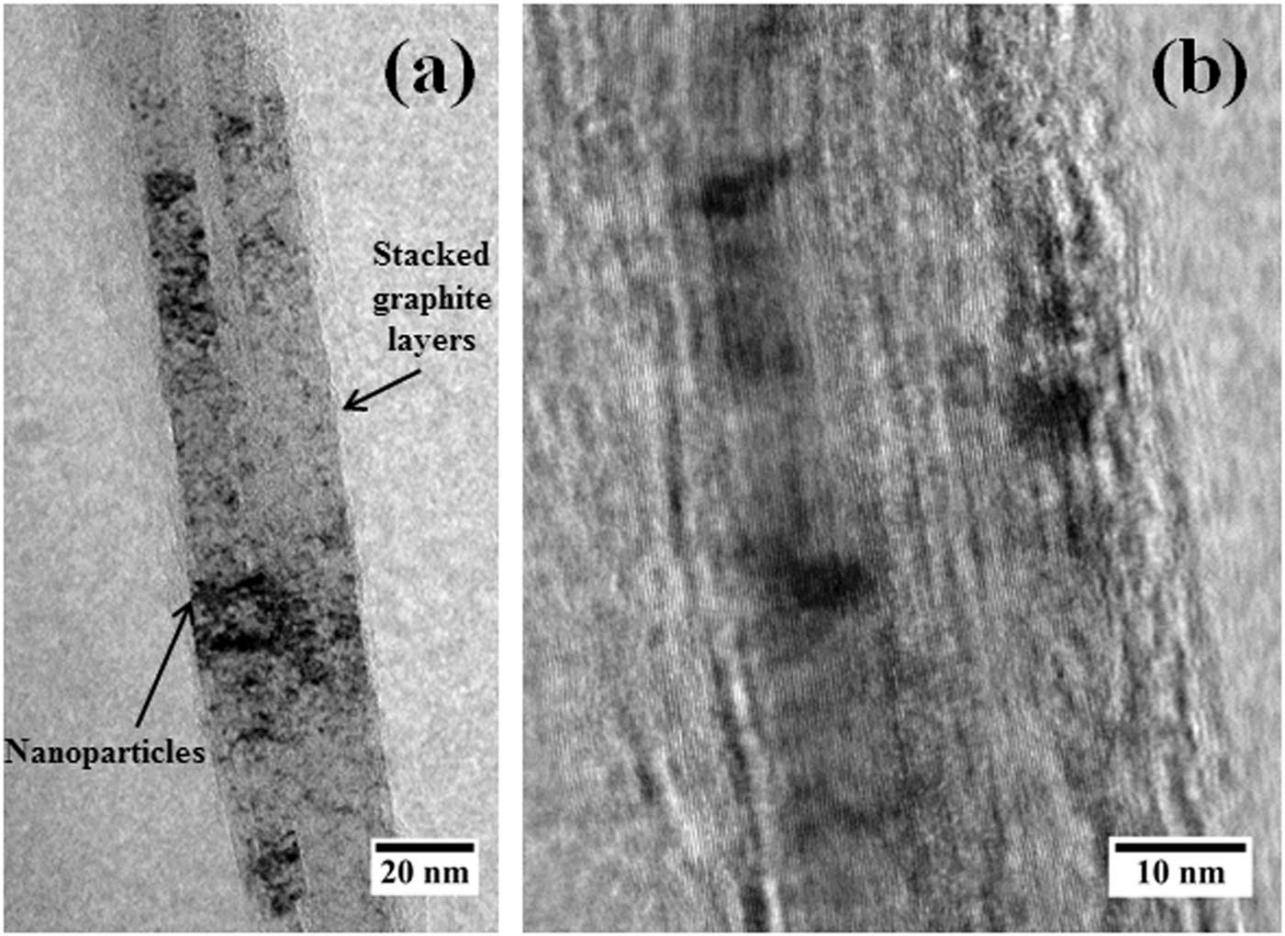
Representative (**a**) low and (**b**) high magnification TEM images of 80 h milled sample showing the stacked graphite layers containing nanoparticles.

SEM micrograph obtained using back scattered electrons from the multimetal-graphite composite after 80 hours of milling is shown in Fig. 1. In Fig. 1, the bright contrast represent the multimetal deposits whereas the dark contrast represents graphite. Figure 1 reveals a fairly uniform distribution of multimetal deposits in the metal-graphite mixture. SEM-EDS compositional analysis revealed the average composition of the multimetal deposits to be 17.14 ± 1.6 at% Fe, 18.59 ± 3.7 at% Cr, 25.83 ± 2.2 at% Co, 31.41 ± 2.8 at% Cu and 7.04 ± 1.1 at% Ni. X-ray diffraction (XRD) peak of the graphite phase obtained from as-mixed and 80 h milled sample is shown in Fig. 2. Considerable shift of the (002) graphite peak to lower 2-theta values reveal a large increase in the interplanar spacing. This indicated towards the incorporation of fine metal clusters within the spaces between the graphite planes during the mechanical milling operation. This was confirmed by TEM imaging of the milled graphite powder. Figure 3(a,b) respectively provides low and high magnification images of the stacked graphene layers (of the graphite) containing ultra-fine particles in the milled sample. Strain caused due to the presence of nanoparticles within the inter-layer spacing facilitated the exfoliation process during the subsequent sonication of the multimetal-graphite powder resulting in the formation of HEA nanoparticle decorated graphene.

**Figure 4:**
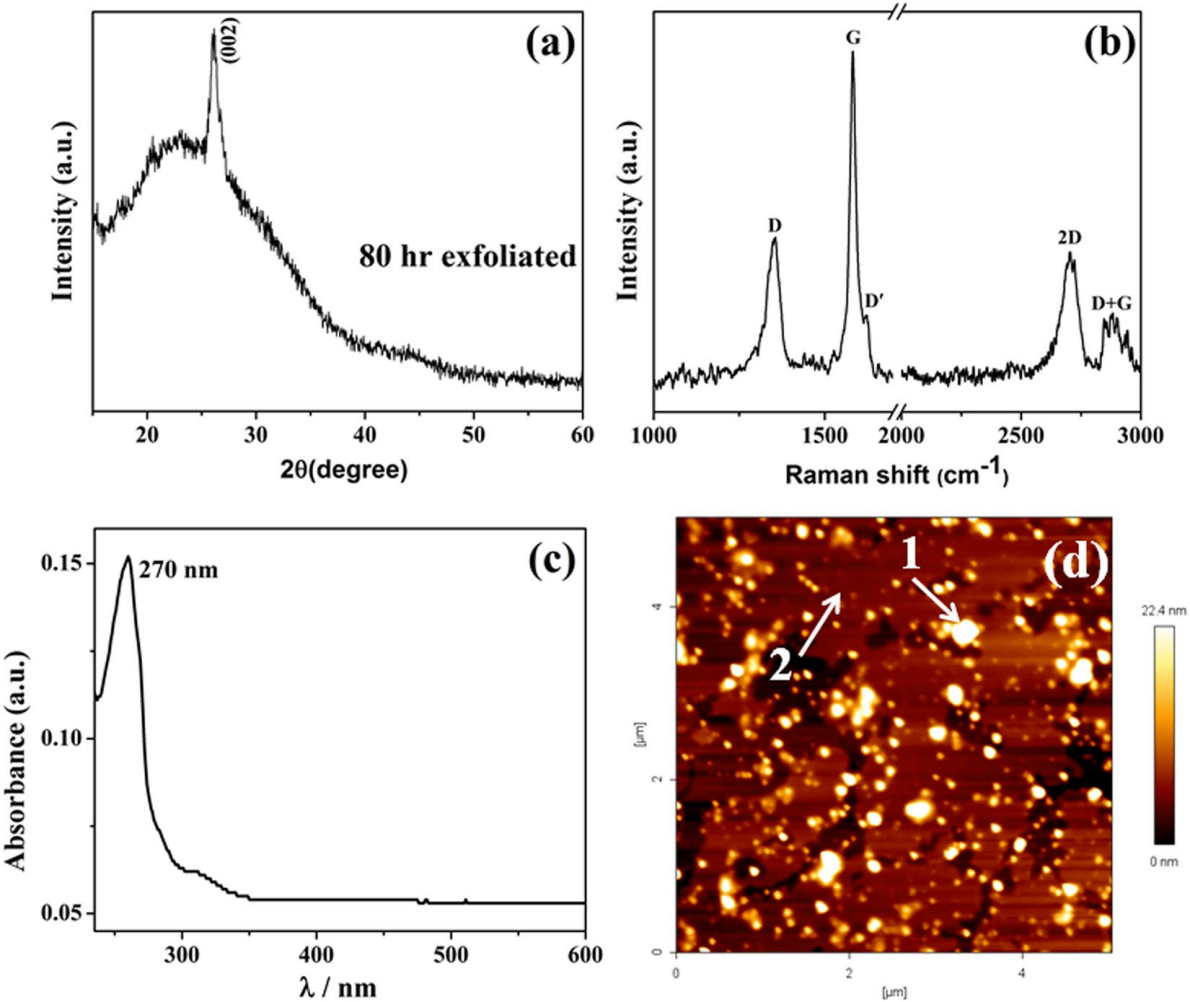
(**a**) XRD profile, (**b**) Raman spectrum, (**c**) UV-Vis absorption spectrum obtained from exfoliated sample, (**d**) representative AFM topographical image of nanoparticle decorated graphene exfoliated sample. Exfoliated graphene (2) and multimetal clusters (1) are denoted by arrows.

The multimetal-graphite composite milled for 80 h was exfoliated using SLS. XRD profile of the exfoliated sample is given in Fig. 4(a). The XRD pattern shows broad diffraction peak at 23.4° 2-theta value and a sharp peak at 26.13° 2-theta value which reveal that the exfoliated sample contains graphene^42^. Raman spectrum obtained from the exfoliated sample is shown in Fig. 4(b). The Raman spectrum shows three characteristic graphene peaks: D band at ~1356 cm^−1^, G band at ~1582 cm^−1^ and 2D band at ~2702 cm^−1^ which corresponds to sp^2^ hybridized carbon atoms. UV-Visible absorption spectrum obtained from the exfoliated sample is provided in Fig. 4(c). In Fig. 4(c), the maximum absorption peak (λ_max_) around 270 nm wavelength which corresponds to the π-π* transition of the aromatic C-C bonds confirmed the presence of graphene in the exfoliated sample^43^. AFM topographical image of the exfoliated sample is shown in Fig. 4(d). Analysis of the Z-height profile from several AFM images of exfoliated samples revealed the average size of the multimetal nanoparticles and the thickness of the exfoliated graphene as 10.3 ± 2.4 nm and 3.3 ± 0.9 nm respectively. The Z-height profile analysis therefore confirmed the presence of nanoparticles and few layered graphene in the exfoliated sample.

**Figure 5:**
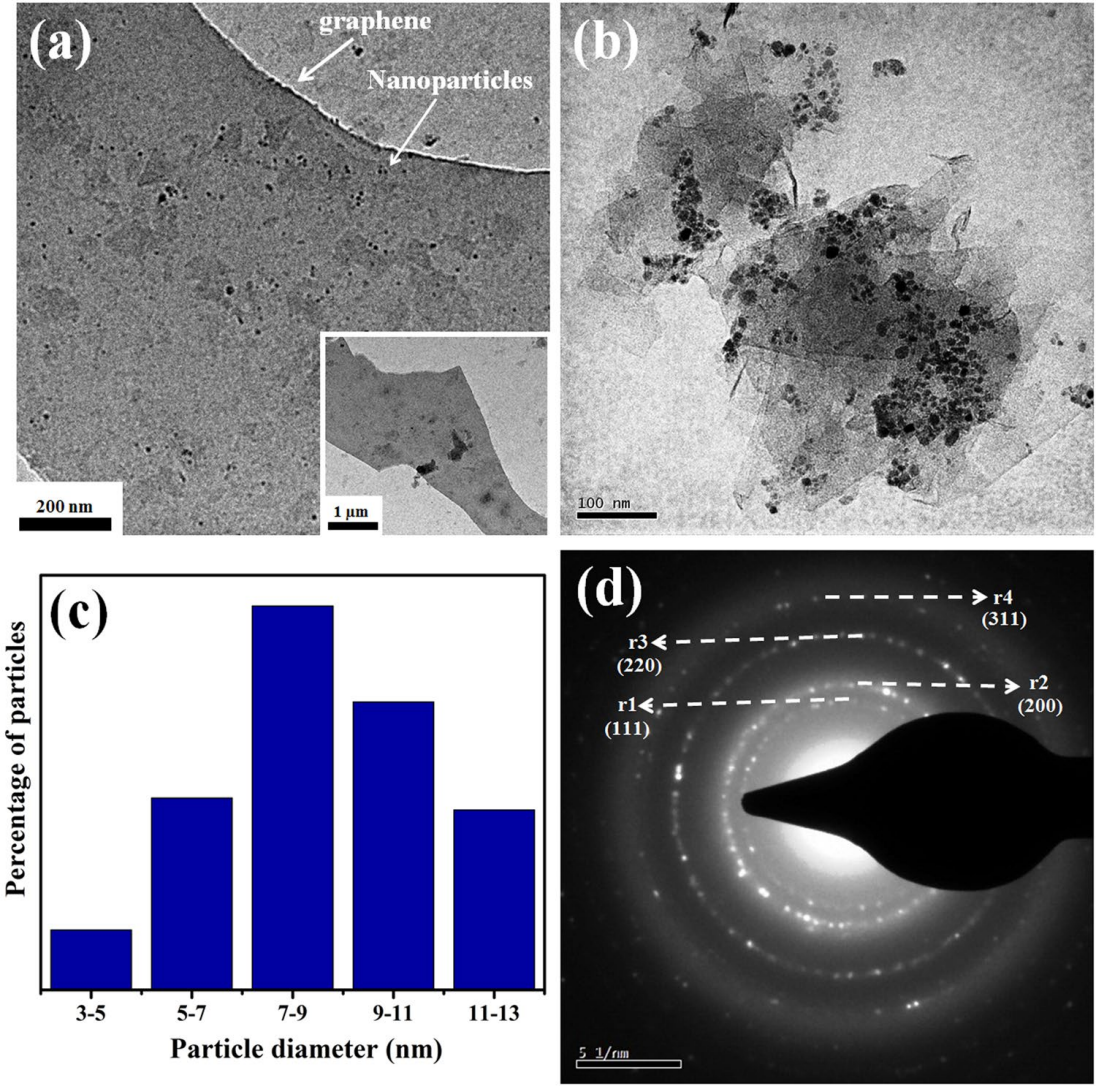
Representative (**a**) low and (**b**) higher magnification image of nanoparticle-graphene sample, (**c**) histogram showing the distribution of nanoparticle sizes and (**d**) SAED pattern obtained from nanoparticle agglomerate. The insert in (**a**) shows the larger graphene sheet from which the main image was obtained.

Representative TEM bright field images of multi-component nanoparticle-graphene composite is provided in Fig. 5(a,b). Figure 5(a) reveals the presence of large graphene sheet with nanoparticles dispersed over it. Figure 5(b) shows nanoparticle-graphene composite in relatively higher magnification. Average size of the nanoparticles calculated from the summation average of sizes of individual nanoparticles was 8 ± 2 nm. The histogram revealing a large distribution in nanoparticle sizes is provided in Fig. 5(c). A representative SAED pattern obtained from graphene decorated with nanoparticles is provided in Fig. 5(d). Interplanar spacing values of the planes corresponding to the diffraction rings in the SAED pattern were calculated as r_1_ = 1.96 Å; r_2_ = 1.72 Å; r_3_ = 1.22 Å; r_4_ = 0.982 Å. Ratio of the d-spacing values (r_1_/r_2_ = 1.14; r_1_/r_3_ = 1.6; r_1_/r_4_ = 1.9; r_2_/r_3_ = 1.4; r_2_/r_4_ = 1.7; r_3_/r_4_ = 1.2) of the diffracting planes matched with the ratio of the d-spacing values of a standard face centred cubic crystal^44^. The lattice parameter of the fcc unit cell was calculated to be 3.394 Å. The SAED pattern did not show diffraction rings corresponding to pure metal phases or their oxides. The electron diffraction analysis therefore clearly revealed alloying of the five component metal atoms in the nanoparticles.

**Figure 6:**
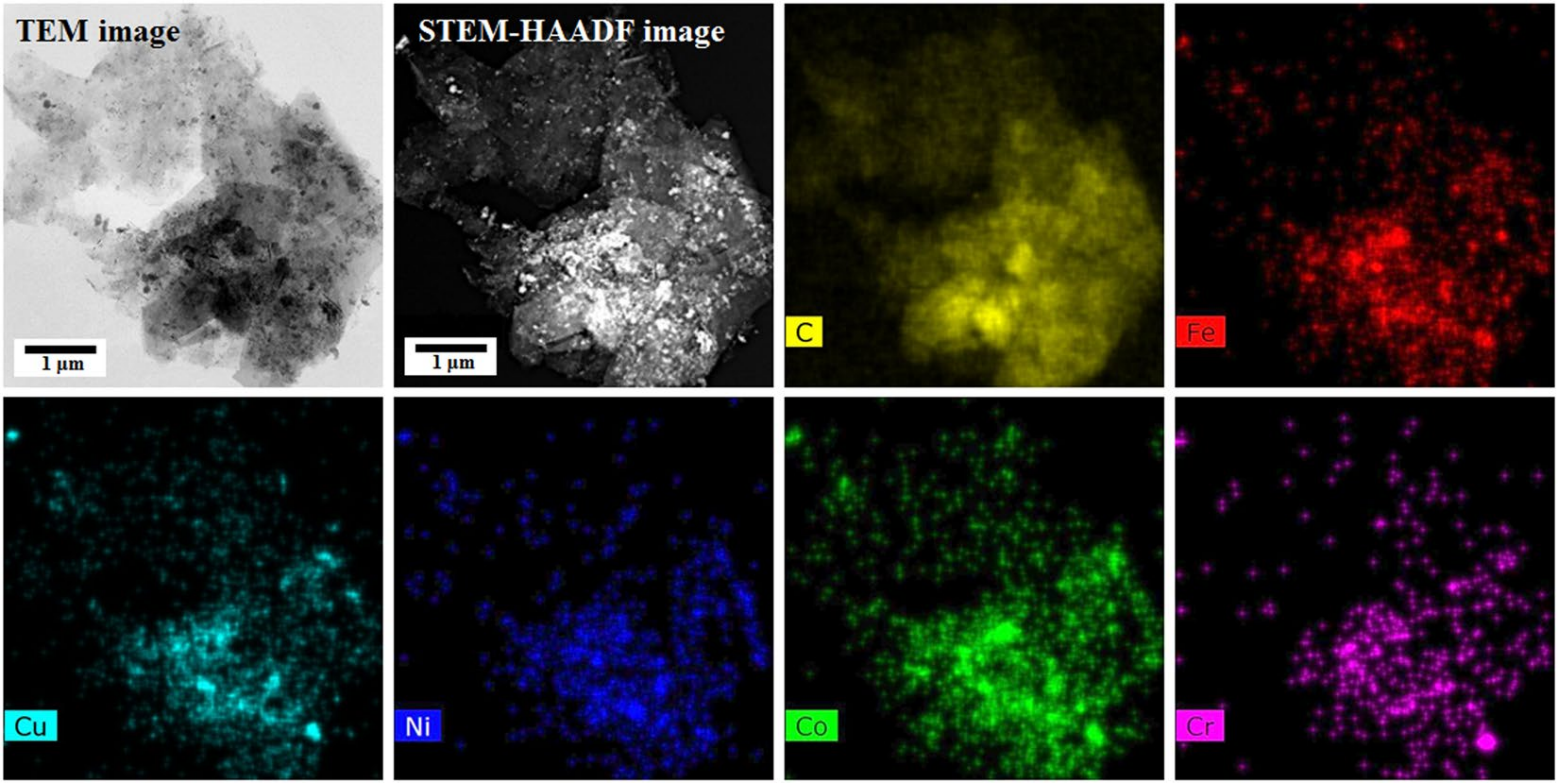
Representative compositional mapping result obtained from the region of interest (nanoparticles over a large graphene sheet).

**Figure 7:**
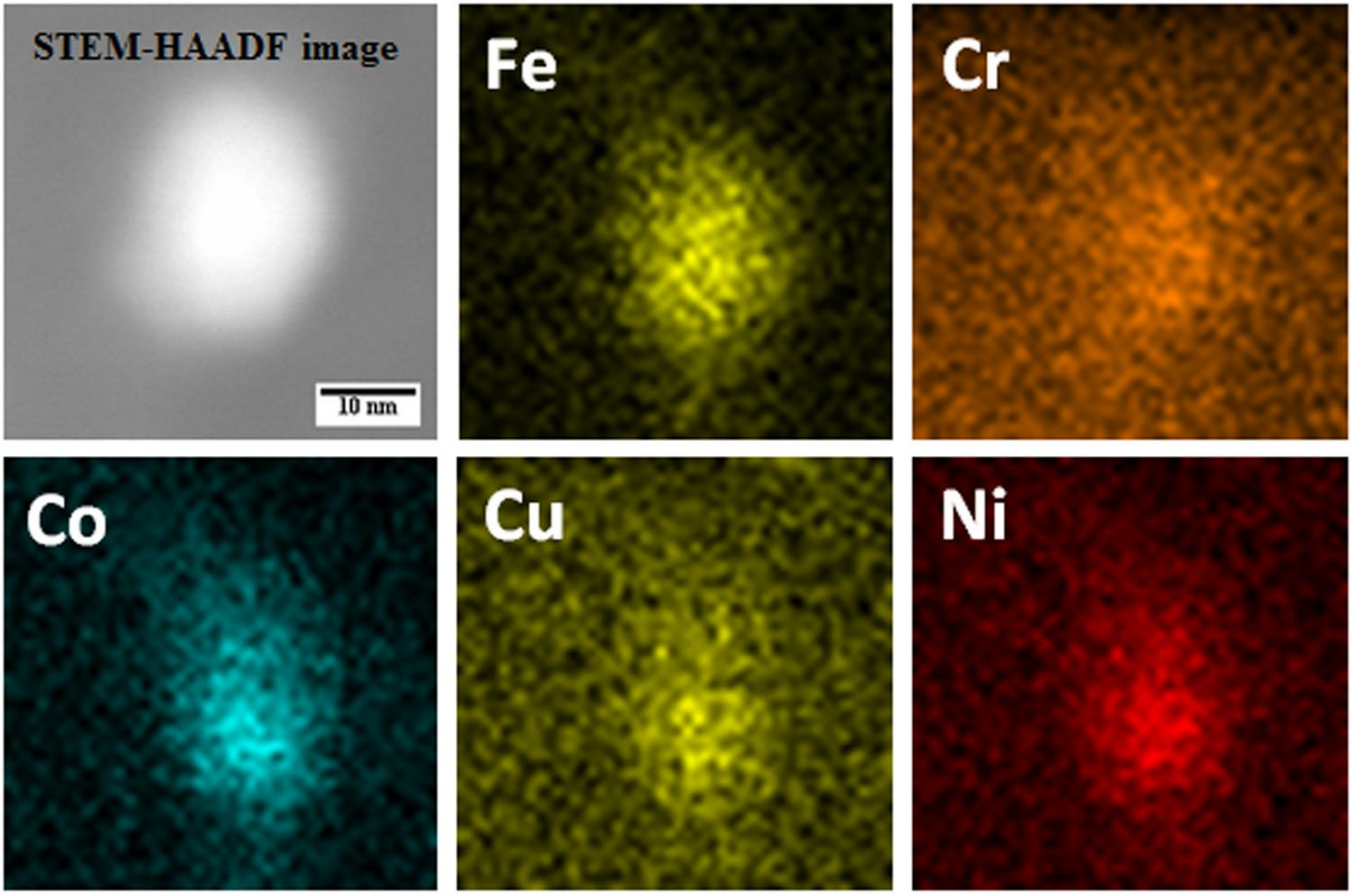
Representative compositional mapping result obtained from a nanoparticle.

**Figure 8:**
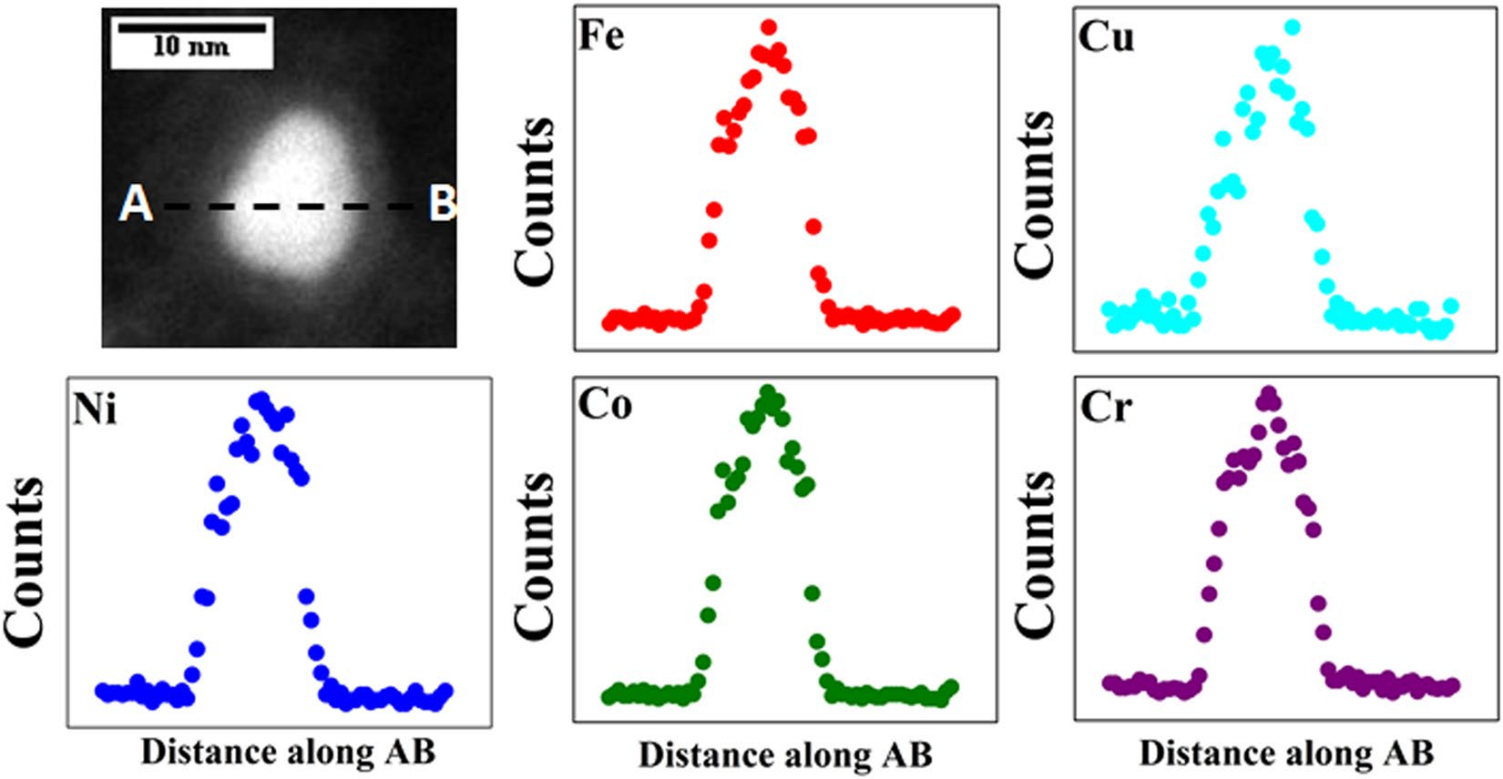
Representative compositional line profile obtained from a nanoparticle.

**Figure 9:**
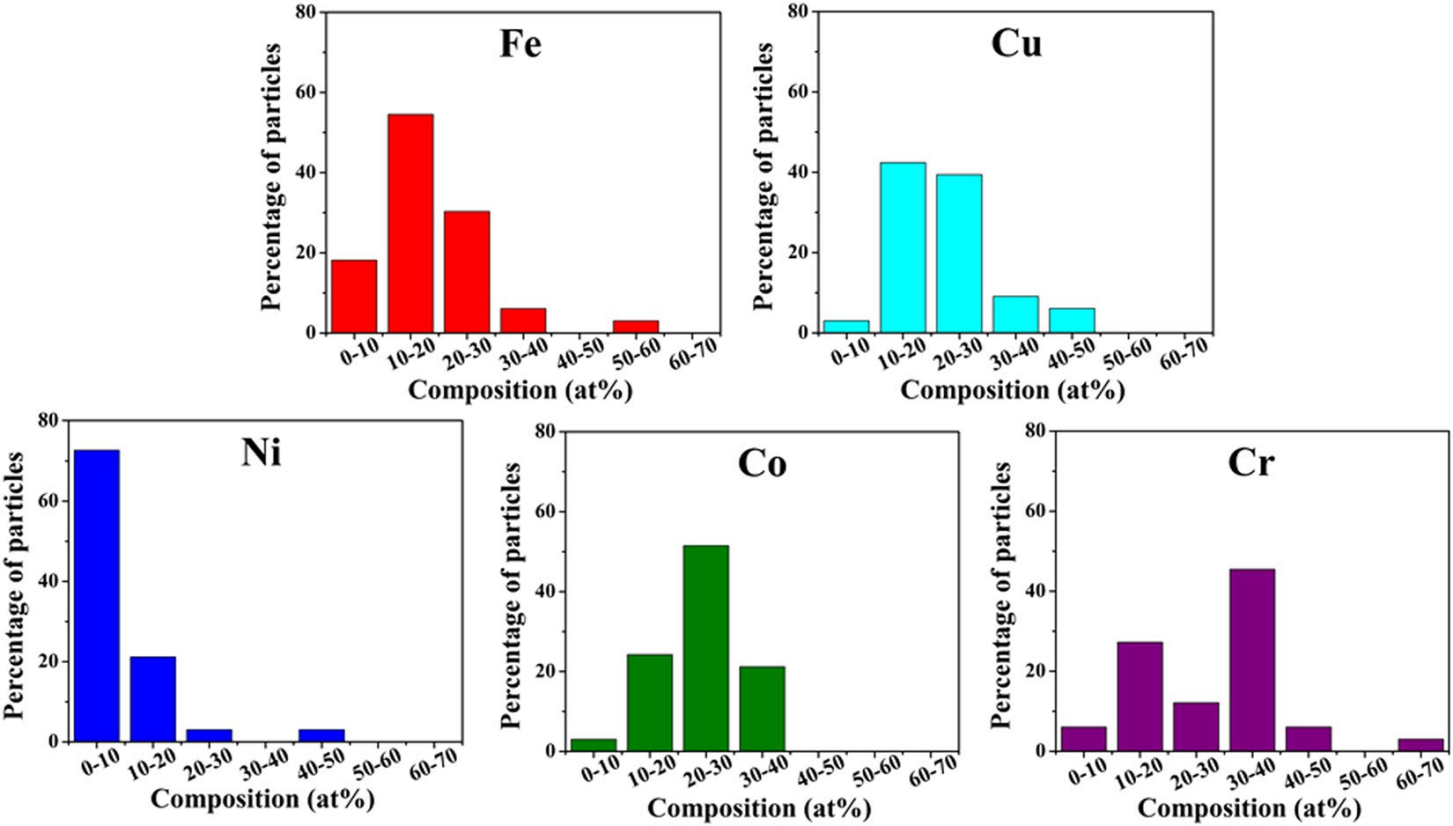
Histogram showing the distribution of five component elements between nanoparticles.

STEM-EDS technique was used for obtaining compositional maps and compositional line profiles. STEM-high angle annular dark field (STEM-HAADF) image of the region of interest (nanoparticles over a large graphene sheet), carbon map, Fe map, Cu map, Ni map, Co map and Cr map provided in Fig. 6 qualitatively shows that the nanoparticles formed over the graphene sheet contains all the five component metal atoms. A representative compositional mapping analysis result obtained from an individual nanoparticle is shown in Fig. 7. A representative compositional line profile analysis result obtained from a different nanoparticle is shown in Fig. 8. It can be observed in Figs 7 and 8 that all the component metal atoms are fairly uniformly distributed within the nanoparticle volume. To investigate the composition distribution between the nanoparticles, elemental composition of 35 individual nanoparticles were determined using the nano-sized electron probe in STEM. Histograms showing the distribution of the five component elements between the nanoparticles is provided in Fig. 9. Histograms in Fig. 9 reveal that all the nanoparticles contain all the five component element atoms however there exists a large distribution in the composition between the nanoparticles. Fe, Cu and Co histograms exhibit similarity in distribution of these elements, Cr histogram shows a relatively wider distribution character for this element and Ni histogram reveal that a large fraction of the nanoparticles contains Ni in the lowest amount among the five component elements.

**Figure 10:**
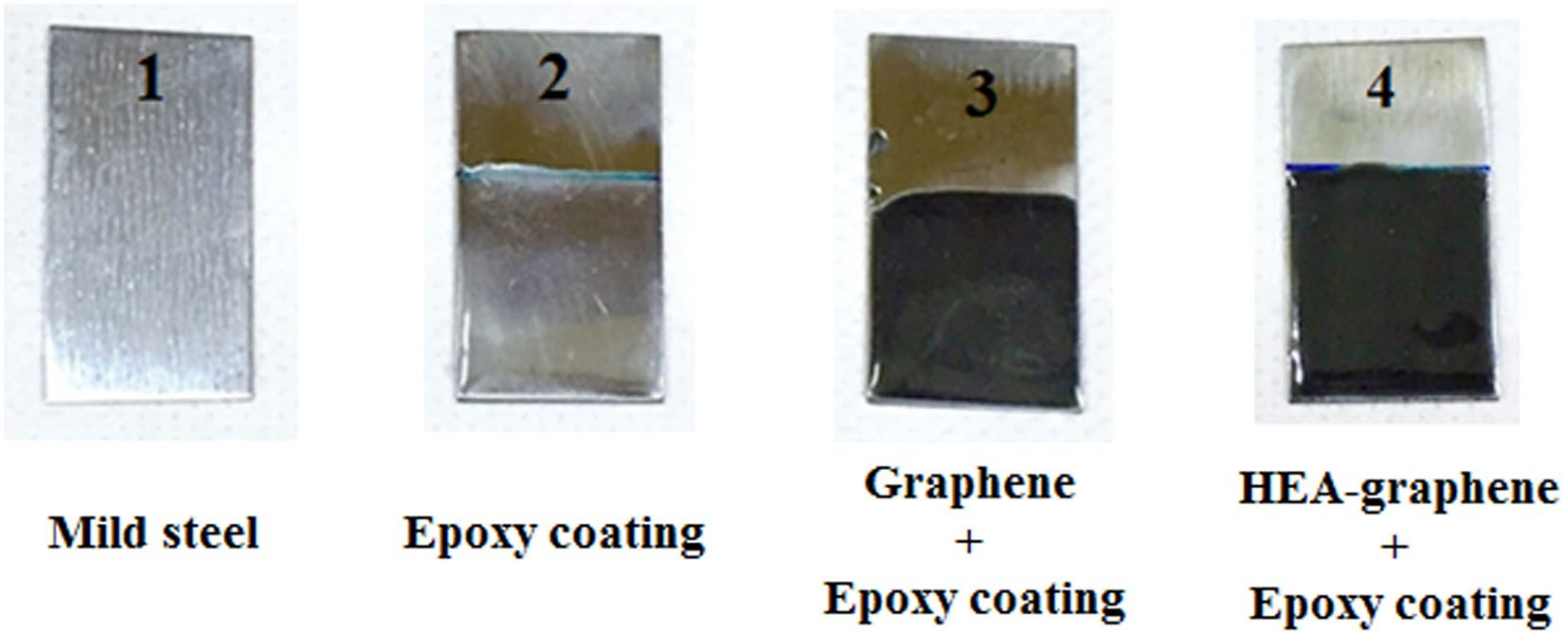
Images of bare mild-steel (MS), epoxy coated MS, graphene-epoxy coated MS and HEA Gr composite-epoxy coated MS substrate.

**Figure 11:**
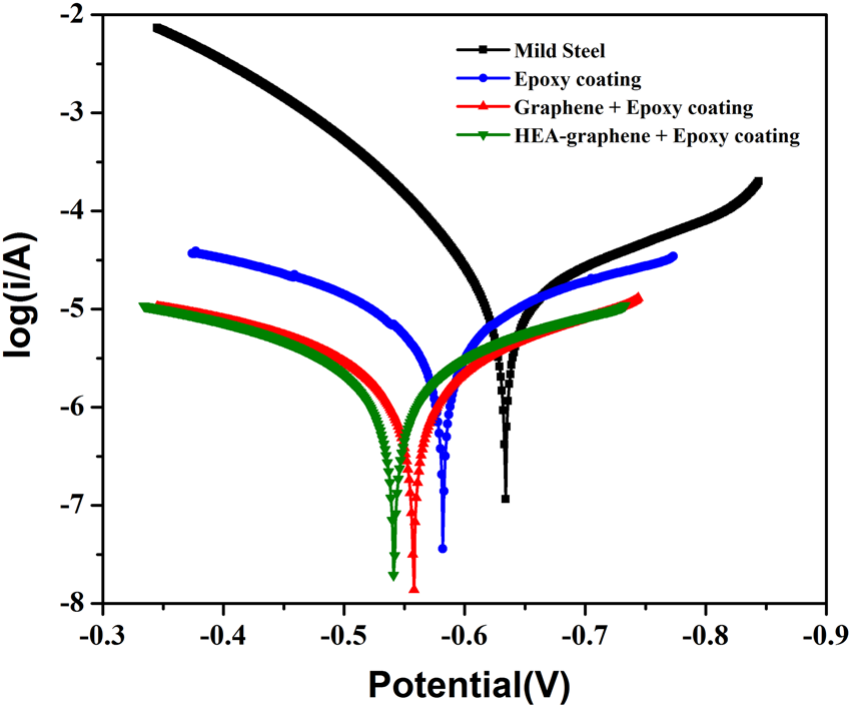
Tafel polarization curves of mild-steel (MS), epoxy coated MS, graphene-epoxy coated MS and HEA Gr composite-epoxy coated MS substrate in 3.5% NaCl solution.

Corrosion properties of as-synthesized multi-component HEA nanoparticle-graphene composite (HEA-Gr) was investigated. HEA-Gr coating was produced by applying a mixture of HEA-Gr composite and araldite standard epoxy adhesive (resin hardener) over mild steel (MS) substrate. Weight ratio of composite to epoxy was 1:40. Corrosion parameters of this coating was compared with the corrosion parameters of bare MS substrate, araldite coated MS and coating (over MS) made by mixing araldite with 80 hours milled and exfoliated only graphene. Figure 10 shows the images of bare MS, epoxy coated MS, graphene-epoxy coated MS and HEA-Gr composite-epoxy coated MS substrate. The electrochemical corrosion analysis was conducted for these samples in 3.5% NaCl solution at room temperature. Tafel polarization or potentiodynamic polarization curves provided in Fig. 11 were measured by polarizing the working electrode (coated sample) to ±200 mV with respect to the open circuit potential (OCP) value at a scan rate of 1 mVs^−1^. The corrosion parameters E_corr_, I_corr_ and corrosion rate (CR) obtained are summarized in Table 1. It can be observed that the HEA-Gr composite-epoxy coating sample exhibits increased E_corr_ value towards positive potential and decreased I_corr_ and CR values when compared to all other samples.

**Table 1:** The corrosion parameters determined from the Tafel plots for mild-steel (MS), epoxy coated over MS, graphene-epoxy coated over MS and HEAs-np Gr composite-epoxy coated over MS substrate.

Sample	Corrosion Parameters		
	E_corr_ (V)	I_corr_ (μA/cm^2^)	Corrosion rate (mil/year/cm^2^)
Mid steel	−0.634	13.76	246.1
Epoxy coating	−0.582	7.34	137.9
Gr + epoxy coating	−0.558	2.16	35.34
Gr:M (80:20) + epoxy coating	−0.541	2.23	26.09

**Figure 12:**
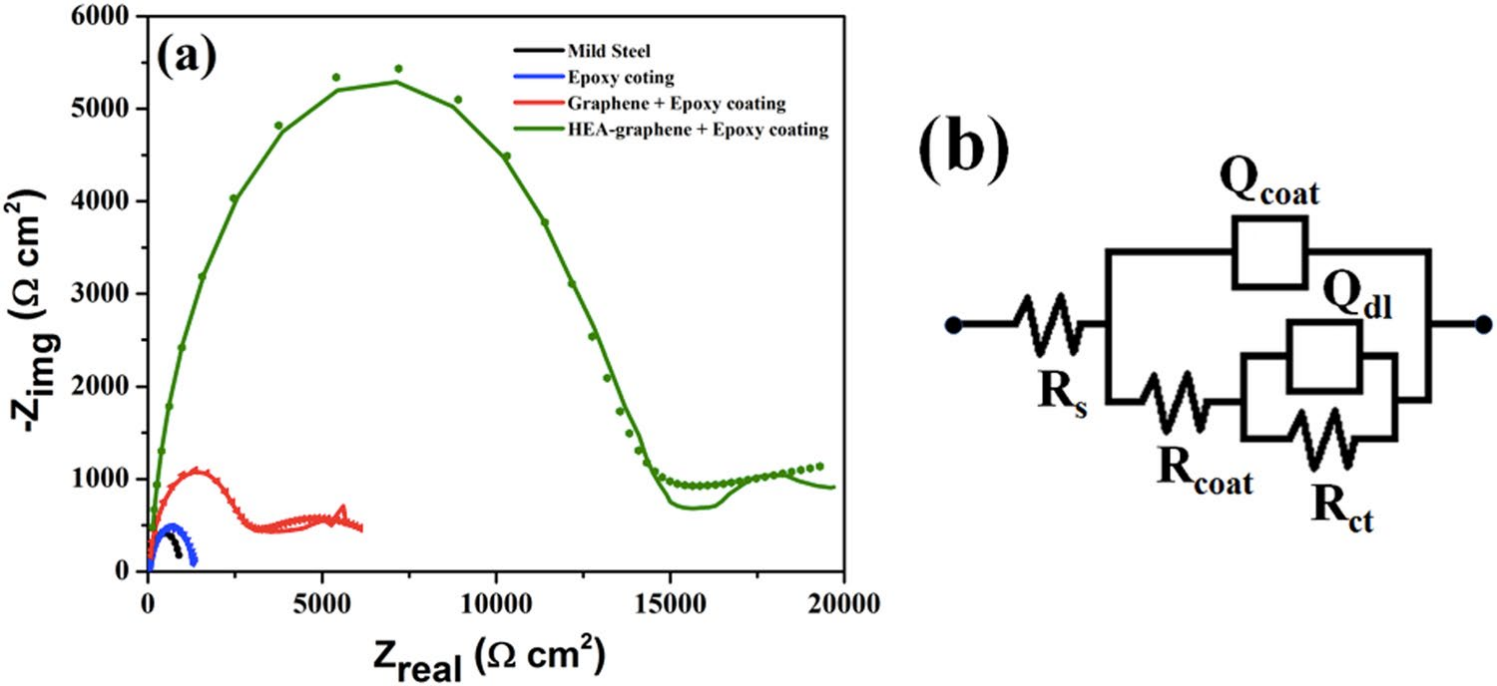
(**a**) Electrochemical impedance spectroscopy (EIS) and (**b**) electrical equivalent circuit (EEC) model of mild-steel (MS), epoxy coated MS, graphene-epoxy coated MS and HEA-Gr composite-epoxy coated MS substrate in 3.5% NaCl solution.

Electrochemical impedance spectroscopy (EIS) were measured at the respective OCP values in the frequency range of 100 kHz to 0.01 Hz with the sinusoidal signal amplitude of 5 mV. The measured EIS data for bare MS, epoxy coated MS, graphene-epoxy coated MS and HEA-Gr composite-epoxy coated MS samples are shown in Fig. 12(a). The EIS plots show two capacitive loops indicating that the corrosion process involved two relaxation events for all the samples. The EIS data were curve fitted with electrical equivalent circuit (EEC) model (using Zsimp win 3.21 software) which is shown in Fig. 12(b). The capacitive element was replaced with a constant phase element (CPE) for better results. In the EEC model, R_s_, R_coat_, Q_coat_, R_ct_ and Q_dl_ are solution resistance, coating resistance, coating capacitance, charge transfer resistance and double layer capacitance respectively. The obtained corrosion parameters associated with EEC for all the samples are provided in Table 2. As observed in Fig. 12(a), the width of the capacitive loop, which is the measure of corrosion resistance or polarization resistance (R_p_) varies significantly between the samples. Total R_p_ i.e. R_p_ = R_coat_ + R_ct_ was found to be 903.9 Ω, 1339 Ω, 5843 Ω and 19650 Ω for bare MS, epoxy coated MS, graphene-epoxy coated MS and HEA-Gr composite-epoxy coated MS substrate samples respectively. Maximum R_p_ value for HEA-Gr composite-epoxy coating represents maximum decrease in the surface activity towards corrosion environment in case of this sample when compared to graphene-epoxy coated, only epoxy coated and bare MS substrate samples. The corrosion parameters illustrate that the presence multi-component nanoparticles over graphene significantly enhances the corrosion resistance offered by only graphene coating. The as-synthesised HEA nanoparticle graphene composite can therefore be spray painted over surfaces to provide enhanced corrosion protection.

**Table 2:** Fitting results for the EIS data acquired from mild-steel (MS), epoxy coated over MS, graphene-epoxy coated over MS and HEAs-np Gr composite-epoxy coated over MS substrate in 3.5% NaCl solution.

Sample	R_s_ (Ω cm^2^)	Q_coat_ (μF cm^−2^)	n (0 < n < 1)	R_coat_ (Ω cm^2^)	Q_dl_ (F cm^−2^)	n (0 < n < 1)	R_ct_ (Ω cm^2^)	χ^2^ square value
Mid steel	1.4	3014	0.93	92.16	225.9	0.8	811.8	3.07 × 10^−3^
Epoxy coating	7.5	539.7	0.8	94	107	0.8	1245	1.36 × 10^−3^
Gr + epoxy coating	28.6	0.738	0.86	2444	16.2	0.76	3399	2.21 × 10^−3^
Gr:M (80:20) + epoxy coating	35.2	0.017	0.9	3914	0.74	0.8	15736	1.1 × 10^−3^

## Conclusion

A two-step method involving mechanical milling and sonication of the milled mixture in the presence of surfactant was used to produce multimetal (Ni, Fe, Cr, Co, Cu) nanoparticle decorated few layer graphene. Composition of the multimetal deposits over graphite after milling was found to be 17.14 ± 1.6 at% Fe, 18.59 ± 3.7 at% Cr, 25.83 ± 2.2 at% Co, 31.41 ± 2.8 at% Cu and 7.04 ± 1.1 at% Ni. This mixture was then dispersed in ethanol containing SLS surfactant. Sonication of this dispersion produced nanoparticle decorated graphene. The nanoparticles were face centred cubic structured. Compositional analysis at the single nanoparticle level revealed that all the five component metal atoms were distributed uniformly within the nanoparticles. A large distribution in composition between the nanoparticles was also observed. The as-synthesised HEA nanoparticle-graphene composite exhibited high corrosion resistance performance making then a suitable materials for corrosion protection application.
